# Antidepressants on Multiple Sclerosis: A Review of *In Vitro* and *In Vivo* Models

**DOI:** 10.3389/fimmu.2021.677879

**Published:** 2021-05-20

**Authors:** Eleni Stamoula, Spyridon Siafis, Ioannis Dardalas, Alexandra Ainatzoglou, Alkis Matsas, Theodoros Athanasiadis, Chrysanthi Sardeli, Konstantinos Stamoulas, Georgios Papazisis

**Affiliations:** ^1^ Department of Clinical Pharmacology, School of Medicine, Aristotle University of Thessaloniki, Thessaloniki, Greece; ^2^ School of Medicine, National and Kapodistrian University of Athens, Athens, Greece; ^3^ School of Medicine, Aristotle University of Thessaloniki, Thessaloniki, Greece

**Keywords:** MS, antidepressants, EAE, neurotransmitters, *in vivo*, *in vitro*, immunomodulation

## Abstract

**Background:**

Increased prevalence of depression has been observed among patients with multiple sclerosis (MS) and correlated with the elevated levels of proinflammatory cytokines and the overall deregulation of monoaminergic neurotransmitters that these patients exhibit. Antidepressants have proved effective not only in treating depression comorbid to MS, but also in alleviating numerous MS symptoms and even minimizing stress-related relapses. Therefore, these agents could prospectively prove beneficial as a complementary MS therapy.

**Objective:**

This review aims at illustrating the underlying mechanisms involved in the beneficial clinical effects of antidepressants observed in MS patients.

**Methods:**

Through a literature search we screened and comparatively assessed papers on the effects of antidepressant use both *in vitro* and *in vivo* MS models, taking into account a number of inclusion and exclusion criteria.

**Results:**

*In vitro* studies indicated that antidepressants promote neural and glial cell viability and differentiation, reduce proinflammatory cytokines and exert neuroprotective activity by eliminating axonal loss. *In vivo* studies confirmed that antidepressants delayed disease onset and alleviated symptoms in Experimental Autoimmune Encephalomyelitis (EAE), the most prevalent animal model of MS. Further, antidepressant agents suppressed inflammation and restrained demyelination by decreasing immune cell infiltration of the CNS.

**Conclusion:**

Antidepressants were efficient in tackling numerous aspects of disease pathophysiology both *in vitro* and *in vivo* models. Given that several antidepressants have already proved effective in clinical trials on MS patients, the inclusion of such agents in the therapeutic arsenal of MS should be seriously considered, following an individualized approach to minimize the adverse events of antidepressants in MS patients.

## Introduction

### Multiple Sclerosis and Depression

Multiple sclerosis (MS) is the most common demyelinating disease of the central nervous system (CNS), involving inflammatory, neurodegenerative and autoimmune patterns in its pathogenesis (1, 2). Most frequently, the onset of MS is characterized by a clinical course of relapses and remissions (RRMS) present in almost 90% of MS patients (3). Current therapeutic means such as disease modifying therapies (DMTs) are mostly efficient during this stage, as CNS inflammation is still highly prominent and directly implied in the emergence of relapses (4, 5). Along with DMTs, antidepressants are often prescribed to MS patients,as they are quite prone to manifest symptoms of depression and anxiety (6–8). In fact, studies report a 50% lifetime risk of major depression for MS patients (9).

### Stress-Related MS Relapses

A significant factor that has been repeatedly held responsible for igniting MS relapses are stressful life events (SLEs) (10, 11). In MS patients, SLEs have proved to spark inflammatory activity by interfering with immune-mediated pathways that regulate autonomic functions, along with the Hypothalamic-Pituitary-Adrenal (HPA) axis (12). Hyper reactivity of the HPA axis is a common finding among MS patients (13). However, chronic stress compromises the ability of endogenous glucocorticoids to regulate inflammation in MS, as it desensitizes immune cells to their regulation by cortisol (12, 14). Resistance to the effects of glucocorticoids has been observed in animals undergoing chronic stress, suggesting that a similar pathway describes the impact of stress on MS patients (15).

### Serotonin and MS

Serotoninergic routes are highly responsible for modulating both our autonomic and neuroendocrine reactions to stressful stimuli, as serotonin constitutes a major HPA axis modulator (16, 17). In patients suffering from depression or anxiety, the serotoninergic network is significantly altered by accumulating stress, thereby severely impacting HPA axis function (18). This defect, however, has proved to be reversed upon antidepressant treatment (19, 20). On that premise, antidepressants could constitute a very promising add-on therapy for MS, as elevated bioavailability of serotonin in MS patients may be efficient in reversing the impact of chronic stress on disease progression.

With respects to serotonin or 5-hydroxytriptamine (5-HT), it displays immunomodulatory properties, interfering with T-cell activation, cytokine release from monocytes, and natural killer (NK) cell stimulation (21–25).Multiple pre-clinical studies have unanimously suggested that selective serotonin reuptake inhibitors (SSRIs) promote remission of the clinical signs of experimental autoimmune encephalomyelitis (EAE), the most prevalent animal model of MS, by curbing pro-inflammatory cytokine release (IFN-γ, TNF-a, IL-6, IL-7) and reducing T-cell proliferation (26–29).

In parallel, solid evidence provided by clinical trials has demonstrated that the use of the SSRI escitalopram in women with MS was effective in preventing stress-related relapses (30). To date, long-term impairment remains the inevitable outcome in most MS cases and current drugs fall short of addressing this fervent matter. It has been proved, however, that long-term disability is highly contingent on the build-up of tokens of impairment that remain after the cessation of each relapse (5). Minimizing relapse frequency is of grave importance for achieving a significant delay in the onset of severe impairment and therefore agents like SSRIs that have proved efficient in this field should be seriously considered as a complementary therapeutic option for all MS patients. Given however the individuality of each MS patient and the varying side events exerted by antidepressants, a personalized prescription of these drugs based on the needs of each patient would be highly recommendable (31).

### Other Key Neurotransmitters in MS

Accumulating evidence suggests that several motor and non-motor symptoms of MS can be attributed to pathologically reduced levels of key neurotransmitters (32–38). Apart from serotonin (39), studies have detected abnormal fluctuations in the levels of noradrenaline (NE) and γ-aminobutyric acid (GABA) (29, 40) within the CNS of EAE mice. Since agents that increase GABAergic and monoaminergic transmission have been shown to moderate EAE severity (29, 41–43), antidepressants could be deemed as potential therapeutic compounds, capable of suppressing the clinical symptoms and neuropathological characteristics of MS (29, 40, 44).

It is worth noting that these key neurotransmitters display both neuronal and immunomodulatory properties, as 5-HT, NE and GABA not only regulate immune cell function (29, 36–38, 45), but also attenuate EAE severity through anti-inflammatory pathways (29, 41, 45). T cells and macrophages express functional receptors and are capable of synthesizing 5-HT, glutamate, GABA and dopamine (DA) (21, 46, 47). Futher, the alpha and beta 2 adrenergic receptors expressed on the surface of T-cells render them susceptible to regulation by adrenergic transmission (48). Similarly, T-cells and macrophages express functional GABA-A receptors, proving that the maintenance of key neurotransmitters at high concentrations is critical for immunomodulation (29, 49).

### Animal Models of MS

As already mentioned, MS is a chronic, autoimmune and demyelinating disease of CNS. While MS is only found in humans, many *in vivo* models have been developed to better simulate the pathophysiology of this disease. None of the *in vivo* MS models is perfect; none of these can reproduce the whole range of complex and diverse morphological and functional aspects of this CNS condition. Each one of them has its advantages and disadvantages, all of them have certain limitations. Albeit certain animal models of MS have proved to be valuable tools, mainly in the development of novel MS drugs (50).

According to a review on MS animal models, the experimental autoimmune encephalomyelitis (EAE) model is one of the most representative *in vivo* MS models as it imitates both the clinical and the pathological characteristics of this condition, followed by the Virus-induced demyelination models (50).

The MS induction on *in vivo* models could be well categorized into three main classes. These include toxin-induced demyelination models, the virus-induced demyelination model mainly by Theiler’s murine encephalomyelitis virus and the above-mentioned widely used experimental autoimmune encephalomyelitis (EAE) model (50, 51).

Toxin-induced demyelination models are based either on linear inoculation of gliotoxins in the white matter, including ethidium bromide (EtBr) and lysolecithin, or on systemically administered toxins, with cuprizone being the most representative. These models offer duplicability, while the demyelinated area is distinct for further remyelinating studies. Furthermore, ethidium bromide, a toxic intercalating agent, affects both the nucleus DNA and the mitochondrial DNA, but offers well established predictable results, as the magnitude of demyelination is concentration-dependent. Lyso-phosphatidylcholine (lysolecithin) has been used for almost 50 years. Its mechanism of action in the demyelinating process is based on its physicochemical properties, as it can act as a detergent-like agent with selectivity over the myelin-producing cells marking and engaging T and B cells, like activated macrophages. This method can also be implemented in non-human primates, while also the demyelination can be performed in a spatiotemporal manner. On the contrary, this method does not lead to any immune response resembling the one recognized during multiple sclerosis (50).

Certain other toxins possess analogous demyelinating toxic results but are not in general use. Examples include ionomycin, a calcium ionophore, 6-aminonicotinamide, an antimetabolite of niacin and diphtheria toxin. Antibody-mediated demyelination is also an acknowledged animal model of induced demyelination by galactocerebroside antibodies. Finally, this class of methods included cuprizone, a copper-chelating agent, which has been shown to be toxic for myelin, affecting both white and grey matter leading to oligodendrocyte apoptosis, mitochondrial enzyme malfunction and activation of microglia. Like lysolecithin, cuprizone can also be performed in a spatiotemporal manner while interest is focused on the combined use of cuprizone with other methods of demyelination induction like EAE.

There is growing indication that certain viruses are involved in the pathogenesis of MS, functioning like environmental triggers. The Epstein-Barr virus (EBV) is a typical example that has long been associated with autoimmune conditions including multiple sclerosis despite the exact cause still remains unknown (51). Viruses that have been used *in vivo* as MS inductors include Theiler’s murine encephalomyelitis virus (TMEV), the canine distemper virus and the mouse hepatitis virus. The former is the most established and serves as a neurotropic viral infection model. TMEV can be separated into two main categories based on the virulence of the viral strains or subgroups and the qualification to induce demyelination. The effects of each viral subgroup extend from severe encephalitis to deadly encephalomyelitis, also being subject to the mouse strains. The most defiant are the BALB/c, C57BL/6J, C57BL/10, and C57/L mouse strains (50). This model can lead to both acute and chronic phase of CNS toxicity, outlined by CNS inflammation and neural apoptosis and affecting the subcortical gray matter, the hippocampus and the basal ganglia.

The most established *in vivo* model of MS is the EAE model which can mimic a broader spectrum of histopathological and immunological expressions of the disease. EAE can be induced *via* two different paths, the active immunization with myelin peptides (52)or the passively or adoptively transferred encephalitogenic T cells (53).

Active EAE requires mice, rats, guinea pigs or nonhuman primates, the use of a myelin-related antigen and concomitant injections of pertussis toxin, leading to activated myelin-specific T cells and encephalitogenic lymphocyte–mediated demyelination. Conversely passive EAE is based on the administration of activated, myelin– specific T cells. Passive EAE evolves faster, does not require any adjuvant and showcases better homogeneity, however its main limitation is that the myelin antigen–specific T cells might not have the desired encephalitogenic capacity, when used *in vivo (* 54).

EAE is also affected by the animal strains or species used. The leading option for animals that can accurately imitate the pathophysiology of MS are mice and rats of different strains including Lewis, Dark Agouti (DA) and Brown Norway (BN). Additionally, non-human primates including common marmosets (Callithrix jacchus) and rhesus monkeys (Macaca mulatta), can also be used for *in vivo* experiments on MS (50).

Therefore, the aim of this review is to provide readers with a useful insight into pre-clinical findings regarding the immunomodulatory effects of antidepressants in *in vivo* and *in vitro* models of MS.

## Methods

### Literature Search

We systematically searched the literature for studies investigating the effects of antidepressants on *in vitro* and *in vivo* models of multiple sclerosis. An electronic database literature search was conducted in PubMed, Cochrane and Scopus from inception through 17 April 2021 to provide us with results from *in vivo* and *in vitro* studies.

The following keywords were used: for *in vivo* studies (experimental autoimmune encephalomyelitis OR EAE) AND (MS OR sclerosis) AND antidepressant; for *in vitro* (*In Vitro* or cell culture) AND (MS or sclerosis) AND antidepressant. Retrieved articles were imported to EndNote. All articles were independently screened for duplicity and eligibility by author ES and ID.

### Inclusion and Exclusion Criteria for *In Vitro* Papers

The inclusion criteria for *in vitro* research were the following: i) original research paper, ii) published in English, iii) use of antidepressant drugs/agents, iv) use of antidepressant agents as a monotherapy or combination treatments.

**Figure 1:**
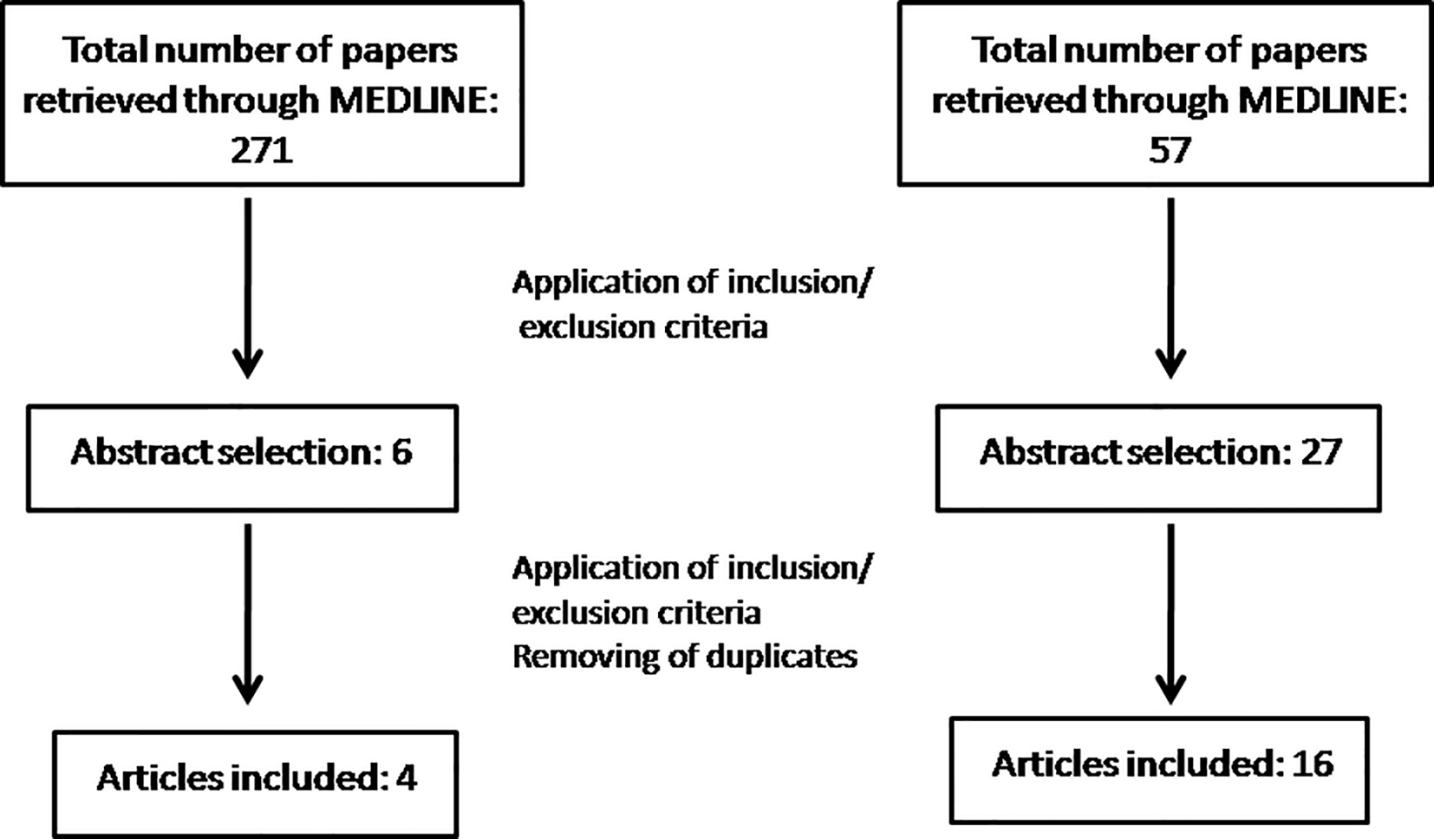
Flow chart of *in vitro* and *in vivo* results.

Articles were excluded if: i) the study did not evaluated MS, ii) the pharmacological agent had antidepressant properties but no clinical use as an antidepressant iv) only the abstract was available, v) the research involved patients. In total, our search yielded 271 articles of which 6 were eligible as abstracts. Finally, after the full text of each article was retrieved and all our inclusion criteria were met, 4 articles were included (**Figure 1**).

### Inclusion and Exclusion Criteria for *In Vivo* Papers

Inclusion criteria for *in vivo* research were the following: i) original research paper, ii) published in English, iii) use of antidepressant drugs/agents, iv) use of antidepressant agents as a monotherapy or combination treatments, v) use of validated *in vivo* tests vi) induction of EAE in mice and rats.

Articles were excluded if i) the study did not evaluated MS, ii) no behavioral tests were used, iii) the pharmacological agent had antidepressant properties but no clinical use as an antidepressant iv) only the abstract was available, v) the article was a review or a case report. In total, our search yielded 59 articles of which 27 were eligible as abstracts. Finally, after the full text of each article was retrieved and all our inclusion criteria were met, 16 articles were included (**Figure 1**).

## Results

### *In Vitro* Results

In our research we ended up with 4 studies on antidepressants use, on *in vitro* models of MS. All studies were performed in *in vivo* and *in vitro* models of MS. Cultures involved cells that were either human or rat and mice derived. Among the drugs examined in this review are the tricyclic antidepressants clomipramine, desipramine, imipramine, amitriptyline, the selective serotonin reuptake inhibitors fluvoxamine (55), and the serotonin- norepinephrine reuptake inhibitor (SNRI) drug venlafaxine (38). The antidepressant effects of these drugs on MS models were evaluated using various methods. Real-time PCR, Western blot analysis and ELISA assay were the most widely used techniques, apart from live-cell imaging, immunohistochemistry, immunostaining and immunofluorescence (IF). Ghareghani et al. found that fluvoxamine enhanced cell proliferation, viability and differentiation of astrocytes, oligodendrocytes and embryonic neural stem cells (eNSCs) (55). Venlafaxine reduced the secretion of pro-inflammatory cytokines such as TNF-a, IFN-γ and IL-6, therefore suppressing inflammation in the CNS, while regulating NK cell and T-cell gene expression (38). Tricyclic antidepressant drugs were found to exhibit neuroprotective activity through elimination of neuronal loss. Reduced proliferation of T-cells and activated B-cells was observed, in tandem with suppression of TNF-a secretion (56).

Ghareghani et al. used murine embryonic neural stem cells from Lewis rat embryos to study the effects of fluvoxamine performing MTT assay to assess cell viability, Real-time PCR, Western blot analysis and Immunofluorescence (IF) analyses. Fluvoxamine was found to act through the Notch signaling pathway, enhancing cell proliferation transcription factors at even low concentrations. Astrocyte, oligodendrocyte and neuron differentiation was observed to be upregulated which may be attributed to upregulation of the mRNA expression of Notch1, Hes1 and Ki-67 (55).

In their study Faissner et al. used cell cultures from both human (brain tissues and peripheral blood mononuclear cells) and murine (splenocytes) origin. Neurotoxicity was induced by rotenone, while HORAC assay, Flow cytometry, live cell imaging, Immunocytochemistry and microscopy were performed. The researchers concluded that Clomipramine, Desipramine, Trimipramine, Imipramine and Doxepin all belonging to the tricyclic antidepressant class, exert beneficial effects in the treatment of MS. Prevention of neuronal loss and antioxidative effects were also observed, while T-cell and activated B-cell proliferation, TNF-a production and plasma membrane compromise were all reduced. These findings highlight an overall neuroprotective activity, that is of pivotal importance for a demyelinating autoimmune disease like MS (56).

### *In Vivo* Results

The *in vivo* results indicated that SSRIs, such as sertraline, fluoxetine and fluvoxamine either delayed disease onset or ameliorated the clinical symptoms in EAE mice. SSRIs mitigated clinical scores and eliminated EAE symptoms, mainly through their actions on immunomodulatory cells. Sertraline-treated mice manifested milder clinical symptoms compared to the untreated EAE group, while sertraline displayed a dose-dependent inhibitory effect on the secretion of the pro-inflammatory cytokines IL-2, TNF-a and INF-γ. Similarly, the reduction of cytokines in mice serum (IL-6, IL-10, TNF-a and INF-γ) was also observed after fluoxetine treatment. Apart from cytokines, fluoxetine also reduced inflammation by directly impacting APC and naïve T-cells. In EAE rats, both fluoxetine (pretreatment/preventive) and fluvoxamine (symptomatic treatment) eliminated clinical symptoms and reduced IFN-γ secretion. Interestingly, fluvoxamine also inhibited the formation of demyelinating plaques, suppressed immune cell infiltration into the CNS and upregulated anti-inflammatory agents. Moreover, in a rat EAE model, duloxetine prevented cold allodynia and showed anti-nociceptive effect on cold hyperalgesia, thus alleviating some clinical signs.

Dose-dependent relief of mechanical allodynia in the bilateral hind paws of EAE mice was also observed after treatment with amitriptyline, a tricyclic antidepressant. In addition, pharmacological intervention with chronic application of amitriptyline in the mild MOG-EAE mice model resulted in a decreased startle reaction and increased hippocampal norepinephrine levels. Another group of researchers (57) utilized the combination treatment or nortriptyline (TCA) and desloratadine (antihistamine) to assess their therapeutic potential on EAE mice. This combination treatment moderated EAE severity by reducing CD4+T cell infiltration in the CNS and suppressing IFN-γ, IL-17 secretion, while boosting anti-inflammatory IL-4 levels. These findings are aligned with other observations supporting that imipramine reduces plasma levels of IL-4 and clomipramine decreases m-RNA expression levels of IFN-γ, TNF-a, IL-17 and chemokine CCL-2. Overproduction of chemokine CCL-5 (also known as RANTES) was mitigated by desipramine, thus restoring glutamate exocytosis and presynaptic cortical defects (57).

In another study, researchers used splenocytes, encephalitogenic T cell clones, primary peritoneal macrophages and brain and spinal cord sections from female mice after the EAE protocol was performed *in vivo*. They conducted ELISA to determine the cytokine levels in the culture supernatants, while carrying out cell viability assay and real-time PCR after RNA isolation. Venlafaxine an SNRI drug was found to regulate the clinical and histopathological impact of EAE. Pro-inflammatory cytokines such as TNF-a, IFN-γ, IL-6, Ccl5 and IL-12 were downregulated while CNS inflammation was also reduced showcasing a potential efficacy in MS (38). According to Dawson et al, fingolimod inhibits the enzyme acid sphingomyelinase sharing a related mechanism of action with desipramine, a tricyclic antidepressant. The researchers used neural-derived cells and fibroblasts and observed that desipramine suppressed ASMase without inducing significant inhibition of other lysosomal hydrolases (58).

According to Taler et al, antidepressants, especially SSRIs, display an immunomodulatory activity by reducing immune cell viability and attenuating of pro-inflammatory cytokine secretion. In particular, their research demonstrated that treatment of EAE mice with sertraline alleviated the neurological symptoms of MOG-induced chronic EAE (42). In addition, fluoxetine suppresses the adaptive immune response in EAE through the reduction of cytokine release (IL-6, IL-10, TNF-a, IFN-γ) and induction of CD4 T-cell apoptosis (59, 60). Recently, a study indicated that the SNRI venlafaxine suppressed the secretion of the pro-inflammatory agents TNF-a, IFN-γ, IL-2 and chemokines in encephalitogenic T cellclones, splenocytes and macrophages, while increasing BDNF expression (38).

Furthermore, treatment of EAE mice with the SNRI venlafaxine ameliorated EAE symptoms in a dose-dependent manner. Venlafaxine exerted its beneficial effects through suppression or enhancement of mRNA expression of proinflammatory and anti-inflammatory factors, respectively. These proinflammatory factors include IFN-γ, TNF-a, IL-12, chemokine CCL-2, CCL-5. On the contrary, venlafaxine increased mRNA expression of the neurotrophic factor BDNF.

Moreover, phenelzine a MAO inhibitor, has been used as a treatment in established EAE- female C57/BL6 mice. It was observed that phenelzine delayed the onset of clinical signs, reduced impairments, ameliorated locomotor function and demonstrated antinociceptive effects. The aforementioned benefits derive from phenelzine’s ability to normalize the levels of GABA and biogenic amines that have been shown to possess anti-inflammatory properties. In particular, phenelzine increased the levels of 5-HT, NE, DA within the spinal cord, brain and brainstem. Lastly, phenelzine normalized pre-synaptic excitatory synaptic densities in S1 and neuronal morphologies.

(**Table 1**, **Table 2**).

**Table 1:** Comparative assessment of *in vitro* studies on the effects of antidepressants in cell and slice cultures.

Ref	Drug	Drug Con.	Cell culture/Slice	Methods	Intracellular signaling/Transcriptional	Results	Comments
					factors		
**Ghareghani et al.** (55)	**Fluvoxamine**	0,1- 1-5-50-100 -500 nM	**Murine eNSCs** (from Lewis rat embryos’ SVZ zone)	MTT assay	*Notch signaling, ↑mRNA expression of Notch, Hes1 and Ki-67, ↑protein levels of NICD*	- ↑ cell viability(0,1-1-5nM)	Flu acts through Notch signaling pathway to enhance cell proliferation
				Real-time PCR		-↑self-renewal capacity of NSCs (neurosphere formation) (1,5, 50nM)	
				Western blot		-Toxic con (500nM) ↑eNSCs differentiation (1 and 5 nM)	
				Neurosphere assay		↑astrocytes and neuron differentiation (5nM)	
						↑oligodendrocyte differentiation (1nM)	
			**Blood samples** (from adult female Lewis EAE rats)	Immunohistochemistry, ELISA		↑IL-4, ↓IFN-γ	
						↓IFN-γ/IL-4 ratio (Th1 indicator)
		0,1- 1-5 nM	**Sections of lumbar spinal cords** (from adult female Lewis rats with EAE)	Neuropathological analysis 17 dpi, quantitative analysis		↓ infiltration of lymphocytes into CNS white matter, ↓inflammatory infiltration with extensive perivascular cuffing, ↓number of infiltrated cells/field	
				GFAP staining, Western blot, HPLC		↓surface areas of demyelination plaques	-Fluvoxamine ameliorates the severity of EAE by inhibiting IFN-γ release and promoting IL-4 production from Th1 and Th2 cells, respectively
						↑MBP in demyelination areas	Fluvoxamine reduces demyelination areas by 0,81%
						GFAP positive staining	Serum lactate is an EAE and MS progression biomarker
						↓serum lactate levels	
**Faissner et al.** (56)	**Clomipramine**	10 μM	**Human neurons** (from brain tissues of therapeutically aborted 15-20 week-old fetuses)	FeSO_4_^-^ - mediated neurotoxicity	*-Chelation with iron*	-Complete prevention of neuronal loss	
				Anti-MAP-2 Ab staining	*-mitochondrial electron transfer chain*	-protective activity	
				Ronetone-induced neurotoxicity		-antioxidative effect even stronger than gallic acid	
				HORAC assay		↓proliferation of T-cells	
		5 μM	**Splenocytes** (from female C57BL/6 mice)	B-cell isolation		↓activated B-cell proliferation	
				FeSO_4_^-^ - mediated neurotoxicity		↓TNF-a production
		2 μM	**PBMCs** (from venous blood from healthy volunteers)	Anti-MAP-2 Ab staining		-strong protection	
				Live-cell imaging	-significant ↓ of plasma membrane compromise (destruction)
	**Desipramine**		**Human neurons** (from brain tissues of therapeutically aborted 15-20 week-old fetuses)	Ronetone-induced neurotoxicity	*-Chelation with iron*	↓proliferation of T-cells	
					*-Propidium iodide leaking inhibition*	↓neurotoxicity
			**Splenocytes** (from female C57BL/6 mice)			↓proliferation of T-cells	
	**Trimipramine**		**Human neurons** (from brain tissues of therapeutically aborted 15-20 week-old fetuses)			↓proliferation of T-cells	
			**Splenocytes** (from female C57BL/6 mice)			↓transcripts encoding IFN-γ, TNF-a, IL-12, Ccl2	
			**Splenocytes** (from female C57BL/6 mice)	Immunohistochemistry PCR			
				LC-MS assay			
				Iba1 staining			
	**Imipramine**		**Spinal cord and cerebellum sections** (from female C57BL/6 mice with EAE)			↓parenchymal inflammation with only a few cells in the meninges	
	**Doxepin**					↓microglial activation and infiltration	
						↓axonal damage	
	**Clomipramine**	25 mg/kg	**Blood samples **(from female C57BL/6 mice with EAE)			Clomipramine serum levels were 751 nM, whereas 28 μM in spinal cord	-High brain to plasma ratio of Clomipramine
**Vollmar et al.** (38)	**Venlafaxine**	10^-4^ to 10^-8^ mol/l	**Encephalitogenic T cell clone 5-8** (MOG 35-55 specific, female SJL/J mice)	Determination of cytokines in culture supernatants by ELISA		↓secretion of TNF-a and IFN-γ	-The effect was more pronounced for IFN-γ and IL-12 p40 with an overall reduction of secretion by 50%
			**Naïve splenocytes** (PLP 139-51 specific, from female SJL/J mice) ->**PLP-specific T cells**			↓secretion of TNF-a, IFN-γ, IL-6, Ccl5, IL-12 p40, ↓secretion of TNF-a and IL-6	-Venlafaxine reduced expression levels of Ccl5, IL-6 and TNF-a dose-dependently
						↓CNS inflammation	-Toxicity observed when concentration of Venlafaxine exceeded 10^-3^mol/l
			**Primary peritoneal macrophages** (activated with LPS, from female SJL/J mice)	Immunohistochemistry –		No reactive gliosis, ↓GFAP gene expression, ↓T cell gene expression (CD3, CD8) in inflamed spinal cord tissue, ↓Granzyme B gene expression in NK cells (in high doses of Venlafaxine)	Venlafaxine reduces the histopathological manifestation of EAE
				GFAP immunostaining			Highest suppressive effect at 60 mg/kg/d
		6-20-60 mg/kg	**Brain and spinal cord sections** (from female SJL/J mice with EAE)			↓IL-12 p40, TNF-a, IFN-γ, ↓transcripts of chemokines Ccl2 and Ccl5, ↑mRNA expression of BDNF (for high doses of Venlafaxine)	Venlafaxine reduces the mRNA expression of inflammation-related genes in spinal cord tissue of EAE mice at day 48 after disease induction
**Dawson et al.** (58)	**Desipramine**	20 μM, 40 μM	**Neural-derived cells** (LA-N-5 and HOG)	Lysosomal hydrolase assay	Displacement of ASMase from the late endosomic/lysosomic membrane	-Inhibition of ASMase	Desipramine reduced ASMase without significant inhibition of other lysosomal hydrolases
				RT-PCR		-No inhibition of β-D-glucosidase	
			**Fibroblasts** (from mouse skin)	Western blot (with anti-ASMase polyclonal Ab)			

Results of *in vitro* papers classified by type and concentration of antidepressant agent, cell or slice culture, methods, intracellular signaling, results and comments.

**Table 2:** Comparative assessment of *in vivo* studies on the effects of antidepressants on disease scores and progression.

Study	Type of antidepressant (SSRI, SNRI, MAO inhibitors)	Dose	Induction of EAE Protocol	Signs of EAE	Preventive or symptomatic treatment	Study Design	(Species) Age/gender/Weight	Methods	Clinical results	Biological results
					(drug administration)					
**Taler et al.** (42)	**(SSRIs)**	5mg/kg	Immunization (SC) with Mog/peptide encompassing amino acids 35-55 of rat	Onset 14/15 dpi and increasing severity 18-25 dpi	7 days after EAE induction	5 groups (10 mice each)	8 weeks old C57/BL female mice Approximately 20g body weight (BW)	Cell viability assay Thymidine incorporation ELISA	Sertraline attenuates neurological symptoms and clinical progression of disease Paroxetine does not affect the clinical score of EAE	↓*ex vivo* viability/proliferation of Mog-activated splenocytes (Ser 0,3μM /Ser 5μM)
	**Sertraline,**				and 3 times weekly for 3 weeks (IP)	I) healthy mice saline treated-controls			sertraline may serve as an add-on option especially in co-morbid major depression	↓pro-inflammatory cytokines (INF-γ, TNF-a, IL-2) from *ex vivo* Mog-Activated EAE splenocytes in a dose-dependent manner (Ser 2,5-30 μM)
	(Paroxetine)					II) EAE mice saline treated				
						III) EAE mice treated sertraline(5mg/kg)				
						IV) EAE treated dexamethasone (1mg/kg)				
						V) EAE treated paroxetine(5mg/kg)				
**Bhat et al.** (59)	**(SSRIs)**	20mg/kg	Immunization (SC) with peptide proteolipid protein PLP 139-151 {100mg PLP 139-151 in emulsion 1:1 with CFA containing 4mg/ml M. Tuberculosis H37Ra	Onset 10 dpi and peaked 13dpi	Once daily/orally	10 per treatment group	8-10 weeks old Female wild type SJL/J mice (*in vivo* treatment) and B10-PL MBP Ac-11 TCR transgenic mice (*in vitro* assays)	ELISA kit Flow cytometry Cell proliferation assay	Decline in mean clinical scores in both groups Fluoxetine delayed onset of EAE and reduced peak illness severity (13-15 days) Ameliorated established EAE	↓immune response (both *in vivo*/*in vitro*)
	**fluoxetine**				I) at the time of immunization	at the time of immunization				↓ cytokines (TNF-a, INF-γ, IL-6, IL-10)
					(delayed-onset model)	I) vehicle group				↓inflammation by directly acting on APC and naive T-cells
					II) at the time of peak disease (day 13)	II) fluoxetine group				↑activation-induced cell death (AICD) (FAS-ligand mediated mechanism)
					(amelioration model)	At the time of peak disease (day 13)				↑CD4-T-cell apoptosis
						I) vehicle group				
						II) fluoxetine group				
**Yuan et al.** (60)	**SSRIs**	10mg/kg	(IP) 200μg of guinea pig spinal cord	Onset of clinical symptoms (piloerection) approximately 4-5 dpi Peak 16 dpi (acute EAE)	Once daily (Fx or saline)	4 groups,	6-8weeks old	ELISA kit	↓ of EAE clinical symptoms (Fx 10/Fx 20)	↓proinflammatory cytokine INF-γ in serum (Fx10 on day 16)
	**Fluoxetine**	20mg/kg			for 14 days prior to immunization	15 per group	Female Wistar rats	Histological analysis	Elimination of inflammatory foci and demyelination in the spinal cord (Fx10)	No difference in serum concentration of TNF-a
	**(pretreatment)**					-Control	160-180 g body weight (BW)		High mortality at dose 20mg/kg	
						-Saline /control				
						(200μl saline intragastric)/				
						-10mg/kg fluoxetine (Fx10)				
						-20mg/kg fluoxetine (Fx20)				
**Thibault et al.** (61)	**SSRIs**	30 mg/kg	EAE induced	Onset of clinical signs 9dpi	Once daily (i.p) after the 14 day post EAE induction	6 groups	5 weeks old female Lewis rats	Actimetry scores	Duloxetine prevented cold allodynia and showed anti-nociceptive effect on cold hyperalgesia (21 to 28 dpi)	
	**Duloxetine**		-solely by MBP			10 per group	150-175 g body weight (BW)	Rotarod (locomotor activity)	Duloxetine relieved cold hyperalgesia on tail region	
			- MBP plus Cyclosporine A (injected subcutaneously three times /week for			-saline		Von Frey test (allodynia/hyperalgesia	Duloxetine does not prevent mechanical hyperalgesia	
			21 days			-EAE + cyclo		Paint-brush test (mechanical allodynia)		
			(1ml CFA/ 4 mg Mycobacterium butyricum/ 500 lg of MBP in			-EAE + cyclo + Acetaminophen		Pinch test (hyperalgesia)		
			0.1 ml of saline)			-EAE + cyclo + Gabapentin		Measure of thermal (cold/heat) allodynia/hyperalgesia		
						-EAE + cyclo + Tramadol				
						-EAE + cyclo + Duloxetine				
**Ghareghani et al.** (55)	**(SSRIs)**	50mg/kg	(SC) 200μl of a 1:1(V/V) mixture of 1g of Guinea Pig Spinal Cord (GPSC) in 1 ml PBS and complete Freud’s adjuvant (CFA) and 1mg/ml enriched M. tuberculosis bacteria	Onset of clinical signs day 12	Treatment initiated (IP) from clinical onset (d 12) for 6 consecutive days(12-17d)	3 groups.	8-12 week old	Immunofluorescent analysis	↓ clinical scores	↓pro-inflammatory cytokine INF-γ in serum
	**Fluvoxamine**				(after immunization)	7 per group	Adult Lewis rats 150-175g body weight (BW)	Western blotting	↓immune cell infiltration into CNS	↑anti-inflammatory IL-4
						- Control (PBS)		HPLC	↓Plaque demyelination (spinal cords)	↑Myelin Basic Protein (MBP)
						-Vehicle (PBS)+ EAE		Histopathological analysis (H/E, LFB)	EAE amelioration	↑glial fibrillary acid protein (GFAP)
						-Fluvoxamine +EAE		Immunohistochemical staining		↓lactate serum levels (MS biomarker)
**Peruga et al.** (62)	**(TCA)**	10mg/kg	(Suboptimal immunization protocol-mild EAE)	Mild motor deficits (tail weakness) 60d.p.i	20 days after immunization	4 groups	10-12 weeks old female C57BL/6 mice	Rotarod	**MOG-EAE mice displayed**:	**MOG-EAE mice displayed**:
	**Amitriptyline**		Immunization (SC) with 50μg MOG _35-55_	(Mild EAE protocol)	Once daily (IP)	I)control/saline (n=5)		Open field	↓exploratory behavior	↓NE and 5-HT
					After 20 days of treatment behavioral analyses were performed			Light/dark box	↑startle reaction	↑TNF-a
						II) control/saline +amitriptyline (n=5)		Startle response	↑LH behavior(depressive-like)	Histopathological alterations in hippocampus
						III) MOG + saline (n=11)		Learned helplessness (LH)	↓neuronal cells	**Amitriptyline treatment꞉**
						IV)MOG + amitriptyline (n=10)		Stereological quantification	**Amitriptyline treatment꞉**	↑norepinephrine level in the hippocampus
								Immunohistochemistry	↓startle response	
								Real-time PCR	↓ anxiety-like and depressive-like behavior	
								HPLC	↓motor impairment	
**Podojil et al.** (57)	**(TCA)**	3mg/kg	Immunization (SC) with 100 μl of an emulsion containing 200μg of or 100 M. Tuberculosis H37Ra and 50μg of PLP_139-151_ or PLP_178-191_in CFA	Onset of remission approximately 15-20 dpi	20 days after immunization	5 groups,10 per group	6-7 weeks old female SJL/J mice	ELISA	High dose of Nortriptyline moderates disease severity	**Combination treatment**
	**Nortriptyline and Nortriptyline + desloratadine (CRx-153)**	5mg/kg	(induction of R-EAE)		Treatment for 21 days via oral gavage	I) vehicle-control		Reversed phase HPLC/MS/MS	**Combination treatment**	[des(3mg/kg) + nor (10mg/kg)]
		10mg/kg				II) desloratadin (3mg/kg)		Delayed type hypersensitivity (DHT) assay	**[des(3mg/kg) + nor (10mg/kg)]**	↓infiltration to the CNS of CD4+ T cells
						III) nortriptyline (3mg/kg)		Flow cytometry	Decrease EAE in SJL/J mice	Alters peripheral T-cell response and cytokine production
						IV) desloratadin (10mg/kg)		Immunohistochemistry	Inhibition of clinical relapses and epitope spreading	Inhibition of Th1 and Th17 differentiation
						V) nortriptyline (10mg/kg)		10-plex LiquiChip (level of cytokines)		Enhancement of Th2 differentiation
						5 groups,10 per group				↓INF-γ, IL-17 (pro-inflammatory)
						I) vehicle-control				↑IL4 (anti-inflammatory)
						II) des(1mg/kg) + nor (5mg/kg)				Dose-dependent decrease in inflammatory cytokines
						III) des(1mg/kg) + nor (10mg/kg)				and alteration in naïve CD4+ differentiation
						IV) des(2mg/kg) + nor (10mg/kg)				
						V) des(3mg/kg) + nor (10mg/kg)				
**Di Prisco et al.** (63)	**(TCA)**	10mg/kg	Immunization (SC) with incomplete Freud’s adjuvant containing M. Tuberculosis 4mg/ml and 200μg of myelin oligodendrocyte glycoprotein MOG _35-55_.	Onset of disease 13+/-1 dpi	Administration of desipramine (dissolved in drinking water) 13 after immunization (acute) or starting from immunization day for 14 consecutive dayschronic)	4 groups.	6-8 weeks female C57BL/6 mice	Rotarod	**Acute treatment:** ↓ neuronal defects and anxiety related behaviors	**Acute treatment**
	**desipramine**				Acute treatment (DMI for 24h on 13d.p.i)	18 per group	18-20g body weight (BW)	Light dark box	**Chronic treatment:** ↓anxiety related behaviors	**(Results at 13 dpi)**
					Chronic treatment (DMI for 14 days)	I) control mice		Open field test	Both treatments (acute/chronic) didn’t improve motor activity or severity of clinical signs	↓overexpression of CCL5 in the cortex of EAE mice
						II) EAE mice		Radioactivity measurement		Long lasting restoration of Glutamate exocytosis and cAMP production (↑cAMP)
						III) Control +DMI (acute)		cAMP -Quantification assay		
						control +DMI (chronic)		ELISA kit		
						IV) EAE mice +DMI (acute)				
						EAE mice +DMI (chronic)				
**Pollak et al.** (64)	**(TCA)**	10mg/kg	Immunization on day 0 and 7 with 300μg MOG	Early onset (day 9) of hyperacute EAE (haEAE) characterized by brain hemorrhage and high mortality rate	Beginning on day 0 mice were either non-handled or injected daily with saline or imipramine	3 groups	female C57BL mice	Observations in motor deficits, food intake, BW, sucrose drinking and social exploration	Imipramine treated group	
	**imipramine**					I) non-handled	4,5-7g body weight (BW)		↑survival rate	
						II) saline			Attenuated haEAE-associated decrease in BW	
						III) imipramine				
**Faissner et al.** (65)	**(TCA)**	25mg/kg	A. Immunization	Onset of clinical signs on 13 day	**Acute EAE-treatment**	**1**-EAE-delayed clomipramine treatment	6-8 weeks female C57BL/6 mice	Flow cytometry	**1**-EAE-delayed clomipramine treatment	**2**-EAE-early clomipramine treatment
	**clomipramine**		(SC) (**C57BL/6** mice)	Onset of clinical signs 18 dpi	**1**-EAE-delayed clomipramine treatment	I)vehicle (PBS) n=8	Approximately 20 g body weight BW	Immunocytochemistry	Disease onset was delayed	↓mRNA expression of INF-γ, TNF-a, IL-17, CCL2
			with 50μg		Initiation of treatment 5 dpi until day 20	II) clomipramine (IP) n=8	8-10 weeks	Microscopy	**2**-EAE-early clomipramine treatment	
			MOG _35-55_		**2**-EAE-early clomipramine treatment	**2**-EAE-early clomipramine treatment	Biozzi ABH mice	Live-cell imaging	Suppression of clinical signs	
			**B. Biozzi ABH**–**EAE**		Initiation of treatment day 0 until day 15	I) vehicle (PBS)(n=8)		Histological analyses	Amelioration of weight loss	
			mouse model (progression model)		**Chronic EAE-treatment**	II) clomipramine (IP) (n=7)		PCR	Attenuation of meningeal inflammation	
			Application of 150 μl emulsion in both sides of hind flanks. Emulsion prepared as follows.		**1**- Treatment initiated at **remission** (days 31 till 42)	Treatment initiated at **remission** (days 31 till 42)		LC-MS	Reduction of microglial activation (less axonal damage)	
					**2**-treatment from clinical **onset** (days 13 till 50)	I) vehicle (PBS), n=10			1-Treatment initiated at **remission** (days 31 till 42)	
					treatment initiated from clinical **onset** (day 18)	II) clomipramine (IP), n=10			No significant difference	
						treatment from clinical **onset** (days 13 till 50)			**2**-treatment from clinical **onset** (days 13 till 50)	
						I) vehicle (PBS)(n=5)			Reduction of clinical severity of the first relapse (days 14-20) and second relapse at late chronic phase (days 42-50)	
						II) clomipramine (IP) (n=6)				
						treatment initiated from clinical **onset** (day 18)			treatment initiated from clinical **onset** (day 18)	
						I) vehicle (PBS) (n=5)			Reduction of clinical severity	
						II) clomipramine (n=5)				
**Vollmar et al.** (38)	**(SNRI)**	6,20,60mg/kg	Immunization (SC) with 200μg proteolipid protein (PLP) 139-151	Onset of clinical signs approximately day 10	Treatment (p.o) initiated at the day of EAE induction	Treatment initiated at EAE induction (oral pretreatment, 14 d treatment) 4 groups (n=8/group)	Age 6-12 weeks	Immunohistochemistry	Treatment initiated at EAE induction (oral pretreatment) (day of adoptive transfer) (14 d): Venlafaxine suppressed EAE in a dose dependent fashion; reduces histopathological manifestation of EAE (20mg/kg) after 3wk treatment.	**Venlafaxine**
	**Venlafaxine**		After *in vitro* restimulation with 10 μg/ml (PLP) 139-151 for 4d, 5*10^6^ to 2*10^7^ cells were injected IP into syngeneic recipients		/or after the onset of clinical symptoms.	I) control (PBS)	Female SJL/J mice	ELISA kit	treatment initiated at the beginning of clinical **onset**: Significant dose dependent reduction of EAE	**(6,60mg/kg) reduces mRNA expression in spinal cord tissue of EAE**
					Control mice received PBS	II) venlafaxine (PO)(6mg)		Cell viability assay	Treatment initiated after manifestation of EAE symptoms: Significant dose dependent amelioration of EAE symptoms after 2wk treatment	↓mRNA expression of CD3 T-cells, cytotoxic CD8 T-cells, Granzyme B
					In addition, in another experiment osmotic pumps were implanted (SC) prior to EAE induction and vehicle or 60mg/kg venlafaxine were administered for 14 consecutive days	III) venlafaxine (PO)(20mg)		Real time PCR	Osmotic pump pretreatment: Reduced peak of disease and ameliorated relapses	↓mRNA expression of pro-inflammatory cytokines
						IV)venlafaxine (PO)(60mg)				INF-γ, TNF-a, IL-12, chemokines Ccl2 and Ccl5
						treatment initiated at the beginning of clinical **onset**, 3 groups (n=10/group)				↑mRNA expression of BDNF
						I) control (PBS)			
						II)venlafaxine (PO)(6mg)			
						III)venlafaxine (PO)(60mg)			
						treatment initiated after manifestation of EAE symptoms, 3 groups (n=10/group)			
						I)control (PBS)			
						II)venlafaxine (PO)(6mg)			
						III)venlafaxine (PO)(60mg)			
						Osmotic pump pretreatment, 2 groups (n=7/group)			
						i)control (PBS)			
						iv)venlafaxine(60mg)			
**Benson et al.** (66)	(MAO-i)	15mg/kg	Subcutaneous 50μg MOG _35-55_	Onset of clinical signs approximately 10-14 d (clinical grade 1)	Treatment	3 groups	10-12 week-old	Open field assays	↓clinical score	↑levels of 5-HT spinal cord (lumbar, thoracic, cervical)
	phenelzine				(IP) initiated from clinical onset (after immunization) and every second day for 14 days(n=14) or daily for 14 consecutive days (n=5)	I) vehicle(saline)+EAE(n=12)	Female C57/BL6	Rotorod assay		↑levels of 5-HT, NE, DA within spinal cord, brain, brainstem
						II) PLZ+EAE(n=14)		HPLC		PLZ treatment every second day causes less inhibition of MAO B
						III) PLZ+EAE(n=5)		Immunocytochemistry		
**Musgrave et al.** (40)	(MAO-i)	15mg/kg	Subcutaneous 50μg MOG _35-55_	Onset of clinical signs day 15 (clinical grade 3)	**Acute** treatment (IP) (PLZ	4 groups	Female C57/BL6	Open field assays	**Daily (chronic)** treatment	**Acute** treatment
	phenelzine	30mg/kg			30mg/kg single dose at the “peak” of disease-clinical score ≥3)	I)control-vehicle (CFA)		Rotorod assay	-Delayed onset of clinical signs	↑levels of 5-HT, NE and GABA in CNS
					**Daily (chronic)** treatment for 28 days (IP) (PLZ	II) control-vehicle (CFA)+ PLZ		HPLC	-reduced impairments	**Daily (chronic)** treatment
					15mg/kg 7 days after immunization)	III) EAE		Histological analysis Immunocytochemistry	-Improved locomotor function	Restores 5-HT levels in the ventral horn
						IV)EAE+ PLZ			-potentiated exploratory behaviors	↑levels of 5-HT, NE in brainstem, cerebellum,
										No difference in GABA
**Potter et al, 2018** (67)	**(MAO-i)**	15mg/kg	Subcutaneous 50μg MOG _35-55_	Onset of clinical signs day 14-17 dpi	Treatment onset 7 days after immunization.	**IHC analysis**	8-12wk old	Rotorod assay	PLZ delayed onset of clinical signs of EAE	PLZ normalized pre-synaptic excitatory synaptic densities in S1; reduced VGLUT1+ density (↓ VGLUT1 reactivity); normalized cortical Iba-1+ reactive microglial cells in S1 (↓excessive cortical Glu release, ↓ cortical microgliosis); normalized neuronal morphologies
	**phenelzine**				**Daily (**IP) injection of either vehicle or phenelzine (15 mg/kg).	I) control (CFA)	Female C57/BL6	FA imaging (FAI)	Chronic PLZ normalized mechanical thresholds in EAE	
						II) vehicle(VEH)+EAE		Von Frey hair assay (mechanical sensitivity)	PLZ demonstrated antinociceptive effect	
						III) PLZ+EAE		Histological analysis		
								Golgi-Cox staining		
								Immunohistochemistry (IHC)		
**Khan et al, 2014** (68)	amitriptyline	1,3 and 7mg/kg	Subcutaneous 200μg MOG _35-55_ mixed with Quillaja sapon. Three different doses of QuilA (15, 30, 45μg) were assessed	Mechanical allodynia in the bilateral hind paws was fully developed by 28-30 dpi	At 30-55 dpi treatment onset with amitriptyline (IP)	Groups	4-6wk old	Histologic analysis	Dose-dependent relief of mechanical allodynia in the bilateral hind paws of EAE mice	
						I) Vehicle	Female C57/BL6	Immunohistochemistry		
						II) EAE + Amitriptyline (1mg/kg)		Von Frey test Gait analysis (automated Catwalk XT)		
						III) EAE + Amitriptyline (3mg/kg)				
						IV) EAE + Amitriptyline (7mg/kg)				
						Sham-mice (n=7)/ EAE-mice (n=32)				
**Stephan et al, 2002** (69)	Imipramine	10mg/kg	Guinea pig MBP (50μg per rat)	Onset of clinical signs	Chronic imipramine pre-treatment (daily via drinking water) started at the age of 6 weeks	4 groups (EAE induction 14wk)	6 week old	Open field test	IMI reversed the increase of deprivation-induced emotionality	↑plasma levels of IL-4
				Control (10-11dpi)	EAE was induced 8 weeks after initiation of the imipramine treatment (postnatal week 14)	Control (undisturbed during 28 postnatal days)	Female Lewis rats	Hole-board test	IMI increased exploration of the hole-board	(protective-like effect of IMI may partly be mediated via TH1 to TH2 shift)
				MD (7-8 dpi)		MD (maternal deprivation for 2h daily for 28d)		ELISA	MD-induced aggravation of EAE is reversed by imipramine	No significant changes of corticosterone, INF-γ and IL-10
				MD+IMI (8-9 dpi)		MD+ IMI (MD for 2h daily for 28d and imipramine treatment initiating 6wk)				
				MD+STIM (5-6 dpi)		MD+STIM (MD plus tactile stimulation for 28d)				

Results of *in vivo* papers classified by type and dose of antidepressant agent, induction protocol and signs of EAE, drug administration, design of study, species, methods, clinical and biological results.

## Discussion

Among MS patients, depression constitutes a highly frequent comorbidity, as studies indicate a 25% prevalence of depression in MS (6, 70). This trend severely affects the quality of life perceived by MS patients, as following disability, depression is the second most impactful factor determining the health-related quality of life (71). Moreover, depression can compromise patient adherence to DMTs, further affecting MS prognosis (72, 73). Although to date, about 86% of depressive MS patients receive antidepressant therapy, depressive symptoms often remain, pointing towards an underdosage or poor matching of these drugs to each patient (74).

Findings encompassed in this review have documented the efficacy of antidepressants in promoting oligodendrocyte maturation and proliferation (55). In MS patients, demyelination is often accompanied by compensatory remyelinating activity, an effect that is principally mediated by oligodendrocyte maturation (75). Therefore, agents like antidepressants or phosphodiesterase inhibitors (76) that stimulate the differentiation of oligodendrocyte precursor cells (OPCs) into mature oligodendrocytes also boost remyelination, thus exerting a neuroprotective effect. This effect can also be indirectly attained through suppression of cytokines that curb Oligodendrogenesis.

The regulation of T cell proliferation and stimulation by antidepressants reported in some studies of this review (38, 56)is of great significance, as these aspects are directly involved in MS pathogenesis. Myelin-reactive T cells are present in MS patients and held accountable for igniting demyelination, therefore the suppression of their activation, proliferation and migration constitute a very salutary property displayed by antidepressants. Lately, the role of B cells in MS has also been described as crucial, involving actions like the orchestration of effector T cell activity through antigen presentation and priming, as well as the secretion of proinflammatory cytokines (77, 78), rendering them principally responsible for the formation of a proinflammatory milieu in the CNS (79).

Studies included in this review also reported the suppression of proinflammatory cytokines induced by antidepressants. Along with several established proinflammatory cytokines such as IL-2, IL-6, IL-12, IL-17, TNFa and IFNγ, antidepressants were also found to reduce serum levels of anti-inflammatory cytokines IL-4 and IL-10, though there has been some evidence supporting some of their immunostimulatory properties (80, 81).

Although MS is considered a Th1 autoimmune disease in which prevails a CD4+ immune response, CD8+ T cells seem to play a pivotal role in the pathogenesis of major depressive disorder (MDD). Clinical studies revealed that CD8+ T cells are increased in MS patients with depression compared to those without, being traceable in their serum during active phases (82). According to other studies, however, CD4+ T cells also seem to be augmented during MDDs in MS (83).

In a clinical scope, antidepressants have proved to be efficient not only in tackling depression comorbid to MS (84, 85), but also even in minimizing stress-related relapses, as shown by the clinical trials of escitalopram on female MS patients (30). Therefore, the use of antidepressants is not only a consolation therapy to improve the quality of life in MS, but also has the potential to significantly modify the course of the disease. Other antidepressants such as vortioxetine combine their antidepressant properties with an enhancing effect on patients’ cognition (86–88). This constitutes a very significant aspect, as about half of MS patients are estimated to manifest cognitive impairment (89). This agent however has neither yet undergone clinical trials on MS patients nor is its efficacy on cognitive enhancement unanimously accepted (90).

Regarding antidepressant use in MS, several adverse events of these drugs could potentially overlap some of the existing deficits that are to be found in MS patients, therefore exacerbating them. To draw an example, SSRIs are known to cause sexual dysfunction, a state that might be already prominent in MS patients, even reaching 85% in female MS patients (91). Therefore, given the heterogeneity of the clinical course of MS in each individual patient, a personalized and patient-oriented approach is necessary to ensure both safety and efficacy in the use of antidepressants in MS (31, 92).

Antidepressants, however, also have the capacity to alleviate numerous MS symptoms. Bupropion can benefit MS patients suffering from chronic fatigue, as this drug has been clinically shown to improve the fatigue severity scale when tested on a patient with MS (93, 94). Fatigue accounts for one of the most prevalent symptoms among MS patients, severely impacting their experienced quality of life. However, the multifactorial and diverse nature of this symptom impedes its management, calling for personalized treatments (95). Therefore, although randomized-controlled trials (RCTs) with numerous participants are required to secure this observation, the identification of a soothing effect of antidepressants on fatigue would constitute a highly significant discovery.

With respect to neuropathic pain, the SNRI duloxetine has been proved to adequately treat this distressing symptom prevalent in more than 25% of MS patients (96), as signified in a double-blind RCT (97). This drug has already received FDA approval for the treatment of peripheral neuropathy in diabetic patients, therefore its inclusion in MS therapy would not be far-fetched. Venlafaxine has also demonstrated some promising qualities regarding neuropathic pain (98), while also tackling the issue of migraines. Although the prevalence of migraines in MS remains unclarified, the importance of their treatment has been repeatedly stressed, as this comorbidity has been correlated with a more symptomatic clinical course of MS (99). Finally, duloxetine has been clinically documented to relieve stress urinary incontinence (100–102), without having yet been tested on MS patients that exhibit this symptom. However, on MS patients suffering from overactive bladder syndrome, a precursor of urinary incontinence, duloxetine was found to be efficient (103).

Taken together, this evidence suggests that antidepressants have proved to be highly effective not only in treating depression in MS patients (85), but also in alleviating numerous distressing symptoms that these patients exhibit (31). Nonetheless, apart from relieving MS comorbidities, antidepressants have even proved to alter disease course and delay progression by curbing stress-related relapses that form a significant pharmacological target in RRMS (30). This clinical background further intensifies the importance of our findings, as basic research studies incorporated in this review unanimously attested to the benefits of antidepressants in MS, both *in vitro* and in the EAE animal model. Regarding *in vivo* MS models, one of the limitations of this review is that it examined only the EAE animal model, which however constitutes the most prevalent and representative animal model currently used in MS research.

However, clinical trials on the matter remain scarce and inconclusive due to the relatively confined number of participants and the uniqueness of each trial, rendering their comparison futile (31). Therefore, clinical testing of antidepressant agents in MS should be further intensified to provide us with reliable assumptions, as existing evidence remains promising.

## Conclusion

All things considered, antidepressants have proved effective both in alleviating EAE, an animal model of MS and *in vitro*, displaying salutary immunomodulatory and anti-inflammatory properties. Clinical studies have also verified the efficacy and safety profile of antidepressants in MS. However, this field warrants further research that would elucidate the underlying mechanisms of action of these agents in MS and highlight their eligibility as a complementary MS therapy.

## Author Contributions

ES: manuscript writing, editing, acquisition of data. ID, SS, AA, AM, TA, KS: Analysis and interpretation of data. CS: manuscript editing. GP: manuscript writing, review of the final manuscript. All authors contributed to the article and approved the submitted version.

## Conflict of Interest

The authors declare that the research was conducted in the absence of any commercial or financial relationships that could be construed as a potential conflict of interest.
